# Incidence and Risk Factors of Ulnar Neuropathy After the Surgical Treatment of Distal Humeral Fractures

**DOI:** 10.7759/cureus.81506

**Published:** 2025-03-31

**Authors:** Masanobu Ogasawara, Hideaki Tanaka, Hiroto Tsukano, Hikaru Tashima, Takuaki Yamamoto

**Affiliations:** 1 Orthopedic Surgery, Fukuoka University Faculty of Medicine, Fukuoka, JPN; 2 Orthopedic Surgery, Kumamoto Orthopaedic Hospital, Kumamoto, JPN

**Keywords:** complication, distal humerus, double plate, fracture, ulnar neuropathy

## Abstract

Background: One of the major complications associated with open reduction and internal fixation (ORIF) for distal humeral fractures is ulnar neuropathy. However, varying reports on its incidence have led to inconsistent opinions. This study aimed to investigate the incidence of ulnar neuropathy in patients who underwent open reduction and internal fixation using two precontoured anatomical locking plates for distal humeral fractures at our hospital. Additionally, we aimed to examine the symptoms and progression of ulnar neuropathy in these cases.

Methods: Among patients who underwent surgery between February 2012 and February 2022, 40 patients with a follow-up period of ≥3 months were included. Fractures were categorized in accordance with the AO Foundation/Orthopaedic Trauma Association (AO/OTA) classification as follows: A2 (n=17), A3 (n=1), C1 (n=17), or C2 (n=5). We retrospectively compared the incidence of postoperative ulnar neuropathy and its symptom severity between Group A (types A2 and A3) and Group C (types C1 and C2). Furthermore, we compared the incidence of neuropathy between groups that did versus did not develop neuropathy.

Results: The incidence of ulnar neuropathy was 35% (14/40 patients), including one patient with concurrent radial neuropathy. Group A had a significantly higher incidence of ulnar neuropathy compared with Group C. Among patients who developed neuropathy, the condition resolved in seven during the study period, while seven remained symptomatic. All patients with persistent symptoms had type C1 fractures. Additionally, a comparison of age between the neuropathy and non-neuropathy groups showed a statistically significant predominance of neuropathy in patients <65 years of age.

Conclusions: Ulnar neuropathy occurred more frequently with distal humeral fractures in patients <65 years of age. Furthermore, the incidence of ulnar neuropathy was significantly higher with intra-articular fractures, suggesting a tendency for persistent symptoms with these fractures.

## Introduction

Distal humeral fractures are challenging and often difficult to treat. The mechanisms of injury range from high-energy trauma in younger individuals to falls in the elderly. Historically, K-wires, 1/3 tubular plates, and reconstruction plates have been associated with high implant failure rates and poor clinical outcomes in distal humeral fractures [1]. However, the advent of anatomical plates and the double-plating method has enabled more robust fixation, leading to improved postoperative outcomes [2-4]. Despite these advancements, ulnar nerve damage remains a common postoperative complication of distal humeral fracture repair. However, the incidence rate varies across reports, and there is no consensus on its interpretation [5-7]. The anterior transposition of the ulnar nerve does not consistently reduce the incidence of nerve damage, and clear risk factors remain unidentified [6-9].

The primary objective of this study is to evaluate the incidence of ulnar neuropathy in patients undergoing open reduction and internal fixation (ORIF) with double plate fixation for distal humeral fractures. The secondary objectives are to identify the risk factors of ulnar neuropathy following surgery and to assess its progression and resolution over time.

## Materials and methods

This retrospective study included patients with a distal humeral fracture of AO Foundation/Orthopaedic Trauma Association (AO/OTA) classification type A or type C. The patients’ medical histories included hypertension, hyperlipidemia, diabetes mellitus, rheumatoid arthritis, cerebral infarction, myocardial infarction, Parkinson’s disease, and schizophrenia; however, none exhibited preoperative impairment in the ulnar nerve territory. All were treated with open reduction and internal fixation using two precontoured anatomical locking plates, placed either orthogonally or in parallel. The Kumamoto Orthopaedic Hospital Research Ethics Examination Committee issued approval 24-10.

**Figure 1:**
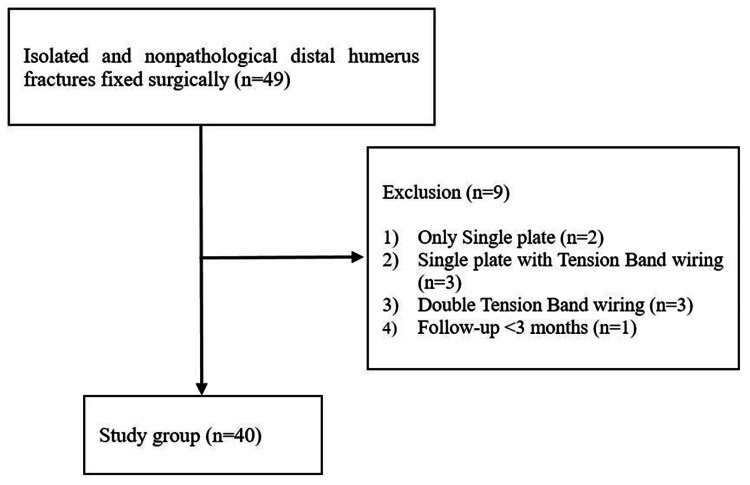
Patient Flowchart

Between February 2012 and February 2022, 49 patients underwent surgical treatment for distal humeral fractures. Of these, two patients were treated with single plate fixation, three with single plate fixation combined with tension band wiring, and three with double tension band wiring. There was one case with a follow-up observation period of less than three months. Surgical procedures with double plate fixation were performed in 40 cases, with a follow-up period of ≥3 months (Figure 1). The mean postoperative follow-up was nine months (range: 3-18 months).

The incidence of neuropathy was evaluated after surgery and at the final follow-up examination. We defined the postoperative period as >48 hours after surgery to differentiate nerve compression symptoms caused by the tourniquet. The potential factors associated with persistent ulnar nerve neuropathy from the postoperative period to the final follow-up examination were also evaluated. Neuropathy was diagnosed by the hand surgeons and defined in accordance with the modified McGowan classification: Grade 1 indicated sensory findings and no motor findings, Grade 2 indicated more severe sensory findings than with Grade 1 and incomplete intrinsic muscle paralysis, and Grade 3 indicated severe sensory findings and intrinsic muscle paralysis [10].

For comparison, patients were divided into neuropathy and non-neuropathy groups. The following parameters were evaluated: sex, age, fracture type, operation time, the anterior transposition of the ulnar nerve, and olecranon osteotomy. Age was categorized as <65 years and ≥65 years, in accordance with a study by Rosenlund et al. [11]. Fracture types were grouped in accordance with the AO/OTA classification, with types A2 and A3 classified as Group A and types C1 and C2 classified as Group C. Surgeries were performed by four different hand surgeons. All surgeries were performed under general anesthesia using a paratricipital approach, with protective handling of the ulnar nerve, and a tourniquet was used in all cases. The operating time was calculated by averaging the duration of all cases and categorized into >3 hours and ≤3 hours.

Statistical analysis was performed using Statcel version 5 (OMS Publishing, Saitama, Japan), which is an add-in for Excel (Microsoft Corp., Redmond, WA). For comparisons of variables with normal distributions, we used the chi-square test. When the sample size was under five, we used Fisher’s exact test. We evaluated sex, age, AO/OTA classification, operation time, the anterior transposition of the ulnar nerve, and olecranon osteotomy. For categories that demonstrated significant differences, multiple regression analysis was performed to identify risk factors associated with ulnar neuropathy. P-values of <0.05 were considered significant.

## Results

The patients’ background information for the neuropathy and non-neuropathy groups is shown in Table 1. The patients’ average age was 67±23.6 years, with eight men and 32 women. The average age in the neuropathy group was 56.3 years, while the average age in the non-neuropathy group was 73.9 years. The mechanism of trauma was a fall from a standing height in 31 cases and a higher-energy injury in nine cases. The breakdown of fracture types by the AO/OTA classification was type A2, 17 cases; type A3, one case; type C1, 17 cases; and type C2, five cases. The anterior transposition of the ulnar nerve was performed in 37 cases and not in the remaining three cases, as the surgeon determined that there was no interference between the plate and the nerve. Among the 12 cases in which olecranon osteotomy was performed, six developed ulnar nerve neuropathy, and two developed radial nerve neuropathy. Orthogonal plating was used in 15 cases, and parallel plating was used in 25 cases. The fixation implant manufacturer was DePuy Synthes (Raynham, MA) in 13 cases, Stryker (Kalamazoo, MI) in 12 cases, Meira Corporation (Nagoya, Japan) in 12 cases, Acumed (Portland, OR) in two cases, and Zimmer Biomet (Warsaw, IN) in one case. In all cases, bone healing was observed, and no implant failures were noted during the healing process.

**Table 1:** Patient Characteristics Olecranon osteotomy: two cases developed radial neuropathy SD, standard deviation; AO, AO Foundation

Variables	Ulnar Neuropathy (n=14)	No Ulnar Neuropathy (n=26)
Sex, male (%)	4 (28.6)	4 (15.3)
Age, median (range)	56.3 (19-82)	73.9 (14-91)
BMI, kg/m^2^, mean (SD)	23.3 (±3.05)	21.5 (±3.55)
Higher-energy injury (%)	4 (28.6)	5 (19.2)
Type of fracture: AO classification		
A2 (%)	3 (21.4)	14 (53.8)
A3 (%)	0 (0)	1 (3.8)
C1 (%)	8 (57.1)	9 (34.6)
C2 (%)	3 (21.4)	2 (7.7)
Operation time, hour, mean (SD)	2.92 (±0.8)	2.85 (±1.19)
Transposition, yes (%)	14 (100)	23 (88)
Olecranon osteotomy, yes (%)	6 (42.9)	4 (15.4)
Plate, parallel plates (%)	9 (64.2)	16 (61.5)

Ulnar nerve neuropathy developed in 14 of the 40 cases, with an incidence rate of 35%. Among these, one case also developed radial nerve neuropathy, and there were an additional two cases with radial nerve neuropathy only. In all neuropathy cases, the symptoms developed immediately after surgery and persisted for more than 48 hours. In accordance with the McGowan classification of neuropathy, there were 12 cases of Grade 1, one case of Grade 2, and one case of Grade 3 [10]. Drug treatment with vitamin B12 was administered exclusively to the case classified as McGowan Grade 3, while the other cases were managed with observation. Symptoms resolved completely in half of the cases, with most symptoms resolving approximately one month postoperatively. Seven of the 14 cases of ulnar neuropathy (Grade 1, six cases; Grade 3, one case) had persistent symptoms. Although most cases involved symptoms that did not interfere with daily life, the Disabilities of the Arm, Shoulder, and Hand (DASH) score was used to evaluate the outcomes. However, the case classified as McGowan Grade 3 showed no improvement in symptoms even after plate removal six months postoperatively. Therefore, the incidence of ulnar neuropathy at the last follow-up in our study was 18%.

Regarding age, 60% of patients aged <65 years and 20% of patients aged ≥65 years developed ulnar nerve neuropathy, indicating that younger to middle-aged adults were significantly more likely to develop the condition. When the fracture types were divided into Group A and Group C, ulnar nerve neuropathy was significantly more common in patients with intra-articular fractures. There were no significant differences related to sex, operation time, anterior transposition, or olecranon osteotomy (Table 2). To identify the risk factors of ulnar neuropathy, multiple regression analysis was performed for age and fracture patterns, which revealed statistically significant differences (P-values=0.04).

**Table 2:** Analysis of the Risk Factors of Ulnar Neuropathy After Distal Humeral Fracture Repair AO: AO Foundation

Variables		Ulnar Neuropathy (n=14)	No Ulnar Neuropathy (n=26)	P-value
Sex	Male	4	4	0.51
	Female	10	22	
Age	<65 years	9	6	<0.01
	≥65 years	5	20	
AO type	Group A	3	15	<0.01
	Group C	11	11	
Operation time	≤3 hours	9	16	0.86
	>3 hours	5	10	
Anterior transposition	Yes	14	23	0.27
	No	0	3	
Olecranon osteotomy	Yes	6	4	0.44
	No	8	22	

## Discussion

The reported incidence of postoperative ulnar nerve neuropathy is 0%-51%, and the exact factors leading to its occurrence remain unclear [6-9,11-16]. The variability in incidence may be due in part to the fact that many previous studies did not use a clear definition of neuropathy [7,9,11-14]. In contrast, the incidence of ulnar neuropathy in our study, using the McGowan classification, was 35%. In comparison, previous studies that used the McGowan classification showed incidence rates ranging from 10% to 51% [5,6,15,16]. Wiggers et al. included only Grade 3 neuropathy in their study, with an incidence rate of 16%, which was higher than the rate in our study (Grade 3: 2.5%) [5]. Vazquez et al. [6] and Oshika et al. [16] included Grade 1-3 neuropathy, but the incidences of neuropathy varied compared with our study because our patients were evaluated both in the postoperative period and at the last follow-up. Even though the same classification was used in these previous studies and our study, it is difficult to simply compare the results because of differences in the time of evaluation, internal fixation devices, and fracture type. The incidence of ulnar neuropathy at the last follow-up in our study was 18%, while Vazquez et al. [6] and Oshika et al. [16] reported incidences of 16% and 29%, respectively, which indicated little variation between the studies. These results indicate that, under the right conditions, the incidence of ulnar neuropathy is approximately 20%, with small fluctuations.

Some authors support the routine performance of ulnar nerve anterior transposition to prevent the development of neuropathy [17,18]. However, Chen et al. reported that anterior transposition does not prevent ulnar neuropathy after distal humeral fracture repair and may instead increase the risk of neuritis [9]. Notably, Svernlöv et al. did not perform anterior nerve transposition in any of their 82 ORIF cases for distal humeral fractures, and there was no significant difference in the incidence of neuropathy compared with ORIF with anterior transposition of the ulnar nerve [15]. A recent randomized controlled trial showed no effect on neuropathy with or without nerve transposition [8]. Similarly, in our study, there was no significant difference in neuropathy with versus without anterior nerve transposition. Notably, there are cases in which anterior transposition is necessary intraoperatively. Yamaguchi et al. performed detailed studies of the vascular anatomy of the ulnar nerve, emphasizing the importance of preserving the nutrient arteries [19]. Moreover, Kurashige et al. suggested that dissecting only the ulnar side of the ulnar nerve while maintaining continuity with the triceps brachii can prevent nerve injury and also mentioned the importance of nutrient arteries [20]. Therefore, routine nerve transposition is unnecessary; however, preserving the nutrient arteries is key to preventing neuropathy when nerve transposition is performed.

As an additional intraoperative maneuver, the effect of olecranon osteotomy on ulnar neuropathy is a concern. In our study, we found no significant difference regarding postoperative neuropathy with versus without this procedure. However, Oshika et al. suggested that olecranon osteotomy could be a preventive factor in the development of ulnar nerve neuropathy [16]. The authors stated that olecranon osteotomy provided sufficient working space to fix intra-articular fragments. It is also easier for the surgeon to visualize the ulnar nerve intraoperatively, when olecranon osteotomy is performed. With AO/OTA classification type C distal humeral fractures, olecranon osteotomy may be necessary, but this is much less common in type A distal humeral fractures. The postoperative risk factors of postoperative ulnar nerve neuropathy after distal humeral fracture repair remain undetermined and are still being investigated.

Our study found a higher incidence of neuropathy in AO/OTA type C distal humeral fractures. Shin and Ring suggested that the combination of trauma and surgical invasion could influence the incidence of ulnar nerve neuropathy (McGowan classification Grade ≥1) [17]. In contrast, Wiggers et al. stated that fracture patterns classified in accordance with the AO/OTA system do not affect the incidence of ulnar nerve neuropathy [5]. The only risk factor was columnar rather than capitellar/trochlear fractures (only McGowan classification Grade 3). These findings suggest that not only surgical factors but also trauma-induced damage to the ulnar nerve may contribute to the development of neuropathy. The mechanism of trauma may be a relevant factor to consider; however, its analysis is challenging, as the magnitude of the applied load varies depending on the patient’s posture, even in similar falls.

Notably, in our study, ulnar neuropathy was significantly more common in people aged <65 years versus ≥65 years. We reviewed similar reports to our study and extracted cases in which double plate fixation was used, for comparison (Table 3) [21-23]. The average age of the neuropathy group across all studies was younger than that of the average age of all patients with proximal humeral fractures. Rosenlund et al., who performed a similar study to ours, showed that the incidence of ulnar neuropathy was approximately 10% higher in patients under 65 years of age [11]. Comparing our results with other reports, it is evident that ulnar nerve neuropathy is more likely in relatively young people. Aguado et al. studied 30 patients over 75 years of age who underwent ORIF with double plate fixation for distal humeral fractures and found that the incidence of ulnar neuropathy was 16%, which was lower than the incidence in our study [24]. Entezari et al. examined the factors associated with radial nerve neuropathy in humeral shaft fractures and reported that the average age of the neuropathy group was eight years younger than that of the non-neuropathy group [25]. These findings indicate that neuropathy tends to occur at a relatively young age.

**Table 3:** Comparative Analysis of Neuropathy Incidence Rates After Distal Humeral Fracture Repair Among Various Research Studies y: years

	Noumi and Tsubo, 2018 [21]	Sogabe et al., 2017 [22]	Iida et al., 2016 [23]	Current Study
Number of cases	11	9	22	40
Number of ulnar neuropathy	3	2	6	14
Incidence rate	27%	22%	27%	35%
Age, median (all cases)	49 y	65 y	74 y	67 y
Age, median (neuropathy)	41 y	51 y	62 y	56 y

On the basis of these results, intra-articular fractures of the distal humerus in relatively young patients are more likely to result in ulnar neuropathy. This is likely owing to the high-energy impact around the elbow joint, which is sufficient to cause intra-articular fractures despite comparatively strong bone quality. Accordingly, it is important to consider various factors, such as age, bone quality, fracture type, the mechanism of injury, and patient background characteristics, when selecting treatment methods and to fully explain the risk of neurological damage to patients.

There are limitations in this study. Notably, this study carries the inherent biases of a retrospective analysis, including potential selection bias associated with the surgeon’s decision to perform anterior transposition. Additionally, the involvement of multiple surgeons in this study may have introduced variations in operative techniques, such as the assessment and preservation of the ulnar nerve’s nutrient vessels. Furthermore, differences in fixation devices could have contributed to the observed outcomes. Another limitation of our study is the short follow-up duration. However, the authors feel that >3 months was sufficient time to detect ulnar symptoms arising from the initial trauma and surgical intervention. The small sample size in this study is also a limitation, as potential differences might become significant with a larger patient population. Prospective studies with comprehensive pre- and postoperative examinations of the ulnar nerve, potentially including electromyographic testing and nerve conduction studies, would likely improve case identification and prevention strategies.

## Conclusions

Postoperative ulnar nerve neuropathy was observed in 14/40 cases (35%) in our study. The risk factors for its occurrence were intra-articular fractures and age of <65 years, which is a relatively young age. Our results suggest that the combination of trauma-induced damage to the ulnar nerve and surgical invasion might lead to neuropathy. Therefore, thorough preoperative explanations and obtaining informed consent from patients regarding the risk of neuropathy are essential.
